# Measurement-device-independent continuous variable entanglement witness in a quantum network

**DOI:** 10.1038/s41377-025-02039-x

**Published:** 2025-11-03

**Authors:** Jing Fu, Xutong Wang, Shengshuai Liu, Jietai Jing

**Affiliations:** 1 State Key Laboratory of Precision Spectroscopy, Joint Institute of Advanced Science and Technology, School of Physics and Electronic Science, East China Normal University, Shanghai, China; 2 Collaborative Innovation Center of Extreme Optics, Shanxi University, Taiyuan, Shanxi China; 3 CAS Center for Excellence in Ultra-intense Laser Science, Shanghai, China

**Keywords:** Quantum optics, Nonlinear optics

## Abstract

The reliable detection of entanglement plays a crucial role in the construction of quantum networks. The conventional entanglement witness (EW) method has high requirements for measurement device and relies on reliable implementation of the measurement. With unreliable measurement device, EW process can be easily attacked by eavesdroppers, which can lead to incorrect entanglement detection results. Therefore, a feasible and secure measurement device independent entanglement witness (MDIEW) method is desired for constructing quantum networks. Here, we detect the continuous variable entanglement by a MDIEW in a quantum network. It is demonstrated that the conventional EW method can be affected when the local oscillator intensity is changed, while the MDIEW method can detect entanglement between users without being affected. Our results provide a trustworthy method to detect entanglement, which is an important step for constructing secure quantum networks.

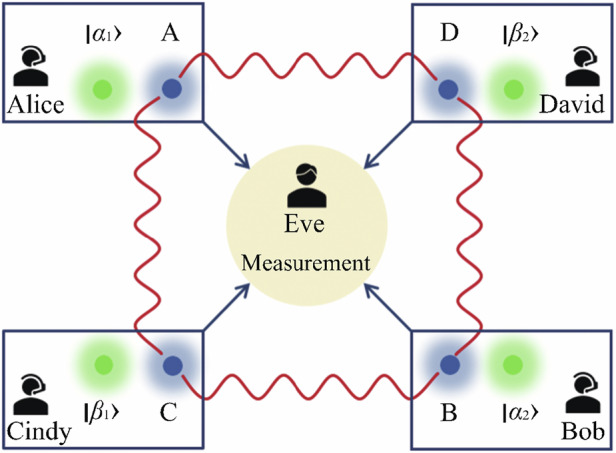

## Abstract (web summary)

We detect the continuous variable entanglement by a measurement-device-independent entanglement witness in a quantum network. Our results provide a trustworthy method of entanglement detection for constructing secure quantum networks.

## Introduction

Quantum networks, which can perform tasks that classical networks cannot perform, play an indispensable role in quantum information processing^1^, such as quantum teleportation network^2,3^ and controlled quantum dense coding network^4^. Quantum entanglement serves as a critical resource for the development of quantum networks^5–13^. The accurate detection of entanglement in quantum networks is crucial for the security of quantum networks. Entanglement witness (EW) is the most common method for detecting entanglement^14^. However, the EW process is easily affected when the measurement device is unreliable. For example, in the continuous variable (CV) quantum system, changing the intensity of the local oscillator (LO) can induce changes in the measured noise power when calibrating shot noise limit (SNL)^15,16^, leading to incorrect entanglement detection results. Therefore, there is an urgent need to find secure and measurement device independent entanglement detection methods.

The measurement device independent entanglement witness (MDIEW) method has been proposed to effectively detect entanglement^17^. This method relies on the use of reliable and well-calibrated coherent states, without the need for reliable measurement device. However, MDIEW has not yet been experimentally implemented in a multi-user CV quantum network. Here we experimentally demonstrate the MDIEW of four pairs of Einstein–Podolsky–Rosen (EPR) entangled modes in a quantum network. Moreover, we construct attacks by changing the intensity of the LO beam. Our results show that the conventional EW method incorrectly identifies separable states as entangled states, while the MDIEW method can accurately distinguish entangled states.

## Results

**Fig. 1:**
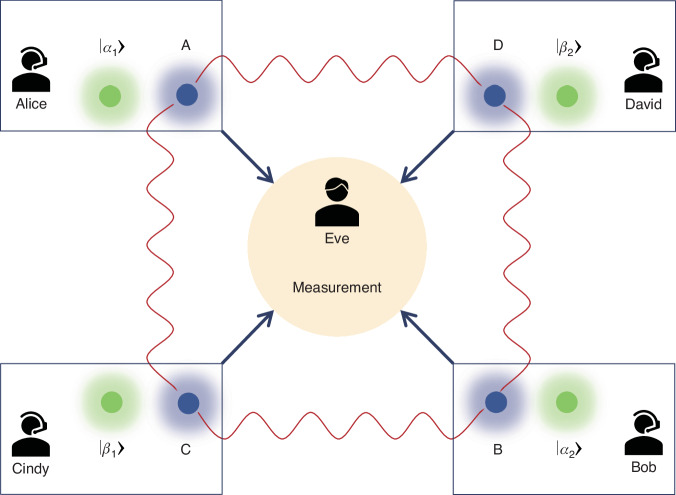
The scheme of the MDIEW in a quantum network. Alice, Bob, Cindy, and David are four users

The measurement device independent nature of the protocol is reflected in the fact that users prepare trusted coherent states independently and the measurements are performed by a third party who can be totally untrusted^17,18^. The schematic diagram of MDIEW in a quantum network is shown in Fig. 1. There are four users, i.e., Alice, Bob, Cindy, and David, in this quantum network. Alice and Cindy (David) each receive a mode from a pair of EPR modes *ρ*_AC_ (*ρ*_AD_). Similarly, Bob and Cindy (David) each receive a mode from a pair of EPR modes *ρ*_BC_ (*ρ*_BD_). Such network is particularly relevant for various quantum communication protocols in CV systems^19,20^.

To detect the entanglement between the obtained modes, each user prepares a trusted and well-calibrated coherent state. Alice (Bob, Cindy, David) prepares a coherent state $\left\vert {\alpha }_{1}\right\rangle$ ($\left\vert {\alpha }_{2}\right\rangle ,\left\vert {\beta }_{1}\right\rangle ,\left\vert {\beta }_{2}\right\rangle$). Then, the users send the prepared coherent states and the received to-be-verified modes to Eve. In the worst case, Eve is an unreliable provider of measurement device. Subsequently, Eve combines coherent states and to-be-verified modes by 50:50 BSs. Then, Eve measures the amplitude and phase quadratures of the BSs’ output modes and announces the measurement results. The four users process Eve’s measurement results based on the information of the coherent states they have prepared. According to the calculated entanglement witness results, users can reliably verify the entanglement between two modes. The above MDIEW process is similar to the measurement device independent quantum key distribution (MDIQKD) protocols where an untrusted relay Eve combines the coherent states prepared by Alice and Bob, performes measurements, and announces the measurement results to Alice and Bob who calculate the secret key^18,21^. The security of MDIQKD protocols is independent of the measurement device and results of the relay^18,21^, and the reliability of MDIEW is also independent of Eve’s measurement results^17^ as the verification relies on the consistency of the measured values by Eve with the prepared coherent state information by Alice, Bob, Cindy, David.

**Fig. 2:**
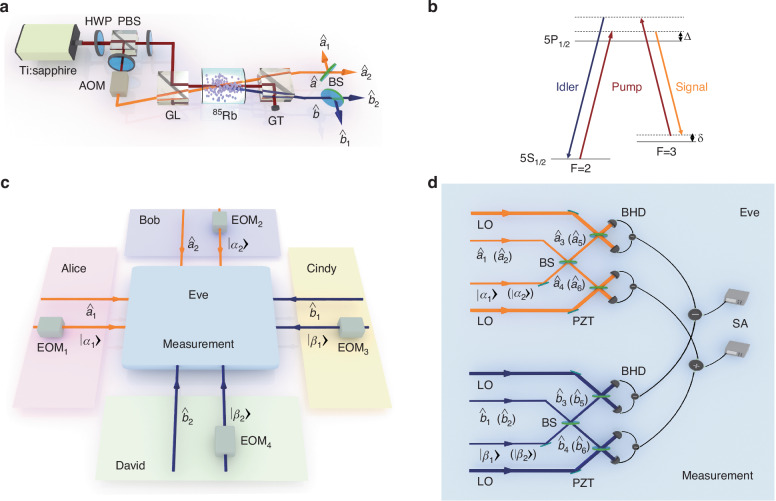
Detailed experimental scheme. **a** The detailed experimental scheme for entanglement resource preparation. PBS polarization beam splitter, HWP half wave plate, GL Glan-Laser polarizer, ^85^Rb ^85^Rb vapor cell, GT Glan-Thompson polarizer, AOM acousto-optic modulator, BS 50:50 beam splitter. **b** Double-*Λ* energy level diagram of ^85^Rb D_1_ line for four-wave mixing process. Δ, one-photon detuning; *δ*, two-photon detuning. **c** The detailed experimental scheme for implementing MDIEW in a quantum network. EOM electro-optic modulator. **d** The measurement of Eve. PZT piezoelectric transducer, LO local oscillator, BHD balanced homodyne detection, SA spectrum analyzer. The balanced detectors have a transimpedance gain of 10^5^ V/A and a quantum efficiency of 97%. The SAs are set to a video bandwidth of 300 Hz and a resolution bandwidth of 300 kHz

The detailed experimental setup for implementing the MDIEW in a quantum network is shown in Fig. 2. The detailed experimental scheme for entanglement resource preparation is shown in Fig. 2a. A cavity-stabilized Ti:sapphire laser emits a Gaussian laser beam with a frequency of about 377.1102 THz. The laser is divided into two beams by a polarization beam splitter (PBS). Passing through a half-wave plate (HWP) and reflected by a Glan laser polarizer (GL), one beam from PBS is seeded into a ^85^Rb vapor cell as a pump beam for the four-wave mixing (FWM) process. The power of this pump beam is about 78 mW. The length of ^85^Rb atomic vapor cell is 12 mm, and its temperature is stabilized at 117 ^∘^C. The other beam from PBS is passed through a HWP and an acousto-optic modulator (AOM) to obtain a signal beam which is 3.04 GHz redshifted from the pump beam. Then, signal beam serves as a seed for the FWM process and crosses with the pump beam at an angle of about 7 mrad at the center of the ^85^Rb vapor cell. As shown in Fig. 2b, in this double-*Λ* configuration FWM process, two pump photons are converted into a signal photon and an idler photon which is 3.04 GHz blueshifted from the pump photon. The Hamiltonian of the FWM process can be expressed as^22^

1 $$\hat{H}=i\hslash \xi {\hat{a}}^{\dagger }{\hat{b}}^{\dagger }+h.c.$$

where ${\hat{a}}^{\dagger }$ and ${\hat{b}}^{\dagger }$ are the creation operators associated with the corresponding optical fields of this process. *ξ* denotes the interaction strength of the FWM process. *h*. *c*. is the Hermitian conjugate. Based on Eq. (1), the relationship between the input and output of the FWM process can be expressed as^22^

2 $$\begin{array}{rcl}\hat{a}(\tau )&=&\sqrt{G}{\hat{a}}_{0}+\sqrt{G-1}{\hat{b}}_{0}^{\dagger }\\ {\hat{b}}^{\dagger }(\tau )&=&\sqrt{G-1}{\hat{a}}_{0}+\sqrt{G}{\hat{b}}_{0}^{\dagger }\end{array}$$

where ${\hat{a}}_{0}$ and ${\hat{b}}_{0}$ are the annihilation operators associated with the seed and the vacuum input, respectively. $G={\cosh }^{2}(\xi \tau )$ is the intensity gain, and *τ* is the interaction time. Based on such FWM process, a pair of EPR entangled modes $\hat{a}$ and $\hat{b}$ are generated^22–27^. Then, $\hat{a}$ ($\hat{b}$) is divided into ${\hat{a}}_{1}$ (${\hat{b}}_{1}$) and ${\hat{a}}_{2}$ (${\hat{b}}_{2}$) by the 50:50 BS. The amplitude quadrature of mode $\hat{o}$ is ${\hat{X}}_{\hat{o}}=(\hat{o}+{\hat{o}}^{\dagger })/2$, and the phase quadrature is ${\hat{P}}_{\hat{o}}=(\hat{o}-{\hat{o}}^{\dagger })/2i$, where $\hat{o}={\hat{a}}_{1},{\hat{a}}_{2},{\hat{b}}_{1},{\hat{b}}_{2}$. In this way, four pairs of EPR entangled modes ({${\hat{a}}_{1},{\hat{b}}_{1}$}, {${\hat{a}}_{1},{\hat{b}}_{2}$}, {${\hat{a}}_{2},{\hat{b}}_{1}$}, and {${\hat{a}}_{2},{\hat{b}}_{2}$}) are constructed. The entangled resources are distributed to users in a quantum network, such as ${\hat{a}}_{1}$ (${\hat{a}}_{2},{\hat{b}}_{1},{\hat{b}}_{2}$) are sent to Alice (Bob, Cindy, David).

**Fig. 3:**
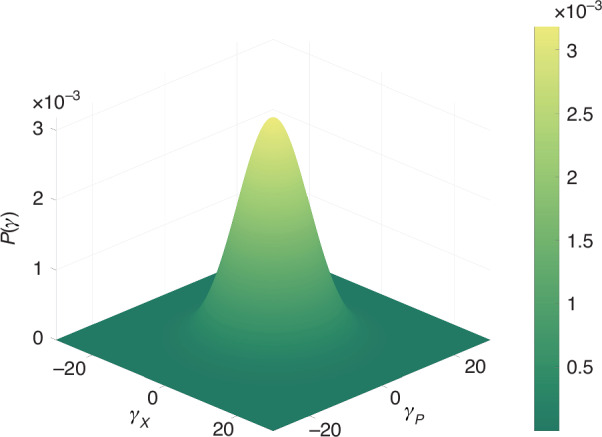
Gaussian probability distribution. Distribution $P(\gamma )=\frac{1}{\pi {\sigma }^{2}}{e}^{\frac{-| \gamma {| }^{2}}{{\sigma }^{2}}}$ is satisfied by the prepared coherent state $\left\vert \gamma \right\rangle$ (*γ* = *α*_1_, *α*_2_, *β*_1_, *β*_2_), where *γ* = *γ*_*X*_ + *iγ*_*P*_ and *σ* = 10

In the quantum network, Alice (Bob, Cindy, David) prepare the trusted coherent state $\left\vert {\alpha }_{1}\right\rangle$ ($\left\vert {\alpha }_{2}\right\rangle ,\left\vert {\beta }_{1}\right\rangle ,\left\vert {\beta }_{2}\right\rangle$), as shown in the pink (purple, yellow, green) area of Fig. 2c. In our experiment, all coherent states $\left\vert \gamma \right\rangle$ (*γ* = *α*_1_, *α*_2_, *β*_1_, *β*_2_) prepared by the users are randomly selected from a Gaussian probability distribution of $P(\gamma )=\frac{1}{\pi {\sigma }^{2}}{e}^{\frac{-| \gamma {| }^{2}}{{\sigma }^{2}}}$ as shown in Fig. 3, where *γ* = *γ*_*X*_ + *iγ*_*P*_ and *σ* = 10 (see-"1. Selection of parameter *σ* value” and Fig. S1 in Supplementary Information for details)^17^. We encode *γ*_*X*_ and *γ*_*P*_ on the amplitude quadrature and phase quadrature through electro-optic modulators, respectively.

As mentioned in Ref. ^28^, for MDIEW, Eve cannot know the information of coherent states in advance. If users utilize only one coherent state for each independent verification, Eve can know this coherent state information after verifying the entanglement once, and adjust the measurement strategy to make users have erroneous judgments on the verified states. Therefore, to avoid Eve knowing the information of coherent states in advance, each independent verification of entanglement requires reselection of coherent states within the Gaussian probability distribution. The reselection of coherent state is enabled by changing the amplitude of the modulation signal, i.e., the values of *γ*_*X*_ and *γ*_*P*_. In other words, parameter *σ* is utilized to control the selection of coherent states, and then the selected coherent states are experimentally encoded by electro-optic modulators.

Alice and Cindy (David) want to verify the entanglement between modes ${\hat{a}}_{1}$ and ${\hat{b}}_{1}$ (${\hat{b}}_{2}$). The tasks of Eve (i.e., measurement) are displayed in the blue area of Fig. 2c. In the MDIEW protocol, Alice sends coherent state $\left\vert {\alpha }_{1}\right\rangle$ and mode ${\hat{a}}_{1}$ to Eve, and Cindy (David) sends $\left\vert {\beta }_{1}\right\rangle$ ($\left\vert {\beta }_{2}\right\rangle$) and mode ${\hat{b}}_{1}$ (${\hat{b}}_{2}$) to Eve. The details of the measurements process are shown in Fig. 2d. Specifically, Eve combines received states by 50:50 BSs and measures outputs modes $\{{\hat{a}}_{3},{\hat{a}}_{4},{\hat{b}}_{3},{\hat{b}}_{4}\}$ ($\{{\hat{a}}_{3},{\hat{a}}_{4},{\hat{b}}_{5},{\hat{b}}_{6}\}$). Piezoelectric transducer (PZT) is placed in the optical path of $\left\vert {\alpha }_{1}\right\rangle$ ($\left\vert {\beta }_{1}\right\rangle ,\left\vert {\beta }_{2}\right\rangle$) to change the relative phase between ${\hat{a}}_{1}$ (${\hat{b}}_{1},{\hat{b}}_{2}$) and $\left\vert {\alpha }_{1}\right\rangle$ ($\left\vert {\beta }_{1}\right\rangle ,\left\vert {\beta }_{2}\right\rangle$). Subsequently, the relative phase between $\{{\hat{a}}_{1},\left\vert {\alpha }_{1}\right\rangle \},\{{\hat{b}}_{1},\left\vert {\beta }_{1}\right\rangle \}$ ($\{{\hat{b}}_{2},\left\vert {\beta }_{2}\right\rangle \}$), are locked to 0 by a micro-control unit^29^, Eve measures the amplitude quadrature ${\hat{X}}_{{\hat{a}}_{3}}=\frac{{\hat{X}}_{{\alpha }_{1}}+{\hat{X}}_{{\hat{a}}_{1}}}{\sqrt{2}}$ (phase quadrature $\left.{\hat{P}}_{{\hat{a}}_{4}}=\frac{{\hat{P}}_{{\alpha }_{1}}-{\hat{P}}_{{\hat{a}}_{1}}}{\sqrt{2}}\right)$ of mode ${\hat{a}}_{3}$ (${\hat{a}}_{4}$) and the amplitude quadrature ${\hat{X}}_{{\hat{b}}_{3}}=\frac{{\hat{X}}_{{\beta }_{1}}+{\hat{X}}_{{\hat{b}}_{1}}}{\sqrt{2}}$ $\left({\rm{phase}}\,{\rm{quadrature}}\,{\hat{P}}_{{\hat{b}}_{4}}=\frac{{\hat{P}}_{{\beta }_{1}}-{\hat{P}}_{{\hat{b}}_{1}}}{\sqrt{2}}\right)$ of mode ${\hat{b}}_{3}$ (${\hat{b}}_{4}$) by phase-locked balanced homodyne detection (BHD), and announces the measurement results to Alice and Cindy. Eve measures the amplitude (phase) quadrature of mode ${\hat{a}}_{3}$ (${\hat{a}}_{4}$) and the amplitude quadrature ${\hat{X}}_{{\hat{b}}_{5}}=\frac{{\hat{X}}_{{\beta }_{2}}+{\hat{X}}_{{\hat{b}}_{2}}}{\sqrt{2}}$ $\left({\rm{phase}}\,{\rm{quadrature}}\,{\hat{P}}_{{\hat{b}}_{6}}=\frac{{\hat{P}}_{{\beta }_{2}}-{\hat{P}}_{{\hat{b}}_{2}}}{\sqrt{2}}\right)$ of mode ${\hat{b}}_{5}$ (${\hat{b}}_{6}$) by BHD, and announces the measurement results to Alice and David. Similarly, Bob and Cindy (David) can verify the entanglement between modes ${\hat{a}}_{2}$ and ${\hat{b}}_{1}$ (${\hat{b}}_{2}$) by the same token. The LO beam is generated by a similar device that is brightly injected and located a few millimeters below the detection beam. The MDIEW expression is^17^

3 $$\left\langle {U}^{2}\right\rangle +\left\langle {V}^{2}\right\rangle \ge \frac{{\sigma }^{2}}{1+{\sigma }^{2}}$$

where *σ* is the aforementioned standard deviation of the Gaussian probability distribution for prepared coherent states^17,30^. For example, when detecting the entanglement between ${\hat{a}}_{1}$ and ${\hat{b}}_{1}$, observables *U* and *V* needed for calculating Eq. (3) can be expressed as

$$\begin{array}{rcl}\left\langle {U}^{2}\right\rangle &=&\left\langle {\left({\hat{X}}_{{\hat{a}}_{3}}-{\hat{X}}_{{\hat{b}}_{3}}-\frac{{\alpha }_{{1}_{{\rm{X}}}}-{\beta }_{{1}_{{\rm{X}}}}}{\sqrt{2}}\right)}^{2}\right\rangle \\ &=&Var({\hat{X}}_{{\hat{a}}_{3}}-{\hat{X}}_{{\hat{b}}_{3}})+{\left\langle {\hat{X}}_{{\hat{a}}_{3}}-{\hat{X}}_{{\hat{b}}_{3}}\right\rangle }^{2}+\\ &&{\left\langle \frac{{\alpha }_{{1}_{X}}-{\beta }_{{1}_{X}}}{\sqrt{2}}\right\rangle }^{2}-2\left\langle {\hat{X}}_{{\hat{a}}_{3}}-{\hat{X}}_{{\hat{b}}_{3}}\right\rangle \left\langle \frac{{\alpha }_{{1}_{X}}-{\beta }_{{1}_{X}}}{\sqrt{2}}\right\rangle \end{array}$$

4 $$\begin{array}{rcl}\left\langle {V}^{2}\right\rangle &=&\left\langle {\left({\hat{P}}_{{\hat{a}}_{4}}+{\hat{P}}_{{\hat{b}}_{4}}-\frac{{\alpha }_{{1}_{P}}+{\beta }_{{1}_{P}}}{\sqrt{2}}\right)}^{2}\right\rangle \\ &=&Var({\hat{P}}_{{\hat{a}}_{4}}+{\hat{P}}_{{\hat{b}}_{4}})+{\left\langle {\hat{P}}_{{\hat{a}}_{4}}+{\hat{P}}_{{\hat{b}}_{4}}\right\rangle }^{2}+\\ &&{\left\langle \frac{{\alpha }_{{1}_{P}}+{\beta }_{{1}_{P}}}{\sqrt{2}}\right\rangle }^{2}-2\left\langle {\hat{P}}_{{\hat{a}}_{4}}+{\hat{P}}_{{\hat{b}}_{4}}\right\rangle \left\langle \frac{{\alpha }_{{1}_{P}}+{\beta }_{{1}_{P}}}{\sqrt{2}}\right\rangle \end{array}$$

Alice and Cindy then process Eve’s measurement results based on the information of the coherent states they have prepared, i.e., calculating the values on the left-hand side of Eq. (3). If Eq. (3) is violated, Alice and Cindy can reliably verify that modes ${\hat{a}}_{1}$ and ${\hat{b}}_{1}$ are entangled. When the quantity $\langle {\hat{X}}_{{\hat{a}}_{3}}-{\hat{X}}_{{\hat{b}}_{3}}\rangle$ ($\langle{\hat{P}}_{{\hat{a}}_{4}}+{\hat{P}}_{{\hat{b}}_{4}}\rangle$), calculated based on Eve’s declared measurement results, does not correspond to the coherent states information $\frac{\left\langle {\alpha }_{{1}_{X}}-{\beta }_{{1}_{X}}\right\rangle }{\sqrt{2}}$ $\left(\frac{\left\langle {\alpha }_{{1}_{P}}+{\beta }_{{1}_{P}}\right\rangle }{\sqrt{2}}\right)$ prepared by Alice and Cindy, the calculated value on the left-hand side of Eq. (3) will be too large to violate Eq. (3). In other words, Eve cannot modify the measurement device to deliberately reduce the left-hand side of Eq. (3), thereby preventing the erroneous identification of separable states as entangled states in the presence of unreliable measurement device and ensuring the reliability of the entanglement witness process^17,31^. Alice and Cindy then can reliably verify the entanglement between two modes through the violation of Eq. (3). In this way, the entanglement verification is performed in a measurement-device-independent manner, as the results depend on the consistency of the measured values by Eve with the coherent states information prepared by Alice, Bob, Cindy, and David, not on the trustworthiness of Eve’s measurement device.

In the context of practical continuous variable quantum key distribution (CVQKD) protocol, the calibration of measurement device has a significant impact on the security of the system. Specifically, a third party can manipulate the measured quadrature by changing the LO intensity^15,16^, thereby affecting the estimated values of various parameters. For example, if the LO intensity is deliberately changed during the calibration of SNL or measuring quadratures, the measured variances of quadratures will not be equal to the actual values, making legitimate partners to incorrectly estimate the secret key rate. The continuous variable MDIQKD protocol is immune to this attack^18^. Similar to the susceptibility of CVQKD protocols to LO intensity attacks, in the case of verifying entanglement, Eve can change the intensity of the LO to cause a change in the measured noise power when calibrating SNL, leading to entanglement detection errors.

**Fig. 4:**
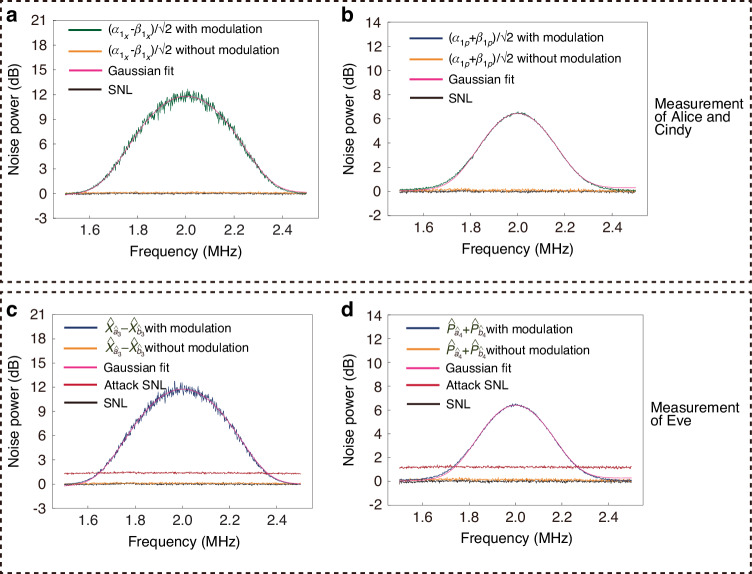
Measurement with unreliable device. **a** and **b** are typical noise power results measured by Alice and Cindy, and **c** and **d** are typical noise power results measured by Eve

We introduce SNL attack by changing the intensity of the LO beam in our scheme. If EPR entangled modes $\hat{a}$ and $\hat{b}$ are blocked, {${\hat{a}}_{1},{\hat{b}}_{1}$}, {${\hat{a}}_{1},{\hat{b}}_{2}$}, {${\hat{a}}_{2},{\hat{b}}_{1}$}, and {${\hat{a}}_{2},{\hat{b}}_{2}$} are separable. The corresponding experimental results of verifying entanglement between ${\hat{a}}_{1}$ and ${\hat{b}}_{1}$ are shown in Fig. 4, in which all black traces at 0 dB represent the normalized SNLs and all red traces represent the “Attack SNLs" when the LO intensity is deliberately increased. The detection is performed at around 2 MHz. The bandwidth of the measured modulation is ~300 kHz (Full Width at Half Maximum). For example, Alice prepares coherent state $\left\vert {\alpha }_{1}=10.476+1.529i\right\rangle$ [*p*(*α*_1_) = 1.038 × 10^−3^], and Cindy prepares coherent state $\left\vert {\beta }_{1}=0.557+0.929i\right\rangle$ [*p*(*β*_1_) = 3.146 × 10^−3^]. It can be seen that these two coherent states are in the corresponding Gaussian probability distribution as shown in Fig. 3. The peak of green trace and orange trace in Fig. 4a (Fig. 4b) at about 2 MHz represent the measurement results of $\frac{{\alpha }_{{1}_{X}}-{\beta }_{{1}_{X}}}{\sqrt{2}}$ ($\frac{{\alpha }_{{1}_{P}}+{\beta }_{{1}_{P}}}{\sqrt{2}}$) for Alice and Cindy with and without modulation signal, respectively, which denote the information of the prepared coherent states. We add Gaussian fit traces (pink traces) to the measurement results with modulation in Fig. 4, allowing the accurate relationship between measured quadratures and the modulation. As shown in Fig. 4a (Fig. 4b), based on pink trace, the noise power of $\frac{{\alpha }_{{1}_{X}}-{\beta }_{{1}_{X}}}{\sqrt{2}}$ ($\frac{{\alpha }_{{1}_{P}}+{\beta }_{{1}_{P}}}{\sqrt{2}}$) for Alice and Cindy with modulated signal is about 11.780 dB (6.455 dB) above the corresponding SNL at 0 dB. Based on orange trace, the noise power of $\frac{{\alpha }_{{1}_{X}}-{\beta }_{{1}_{X}}}{\sqrt{2}}$ ($\frac{{\alpha }_{{1}_{P}}+{\beta }_{{1}_{P}}}{\sqrt{2}}$) for Alice and Cindy without modulated signal is about 0.107 dB (0.126 dB) above the corresponding SNL at 0 dB. In our scheme, SNL$=\langle {\Delta }^{2}({\hat{X}}_{\hat{a}}-{\hat{X}}_{\hat{b}})\rangle =\langle {\Delta }^{2}({\hat{P}}_{\hat{a}}+{\hat{P}}_{\hat{b}})\rangle =0.500$. Therefore, the value of $\frac{\left\langle {\alpha }_{{1}_{X}}-{\beta }_{{1}_{X}}\right\rangle }{\sqrt{2}}$ $\left(\frac{\left\langle {\alpha }_{{1}_{P}}+{\beta }_{{1}_{P}}\right\rangle }{\sqrt{2}}\right)$ can be calculated as $\frac{1{0}^{\frac{11.780}{10}}-1{0}^{\frac{0.107}{10}}}{1{0}^{\frac{0}{10}}}\times 0.500\approx 7.021$ ($\frac{1{0}^{\frac{6.455}{10}}-1{0}^{\frac{0.126}{10}}}{1{0}^{\frac{0}{10}}}\times 0.500\approx 1.696$). The peak of blue trace and orange trace in Fig. 4c (Fig. 4d) at about 2 MHz represent the measurement results of ${\hat{X}}_{{\hat{a}}_{3}}-{\hat{X}}_{{\hat{b}}_{3}}$ (${\hat{P}}_{{\hat{a}}_{4}}+{\hat{P}}_{{\hat{b}}_{4}}$) for Eve with and without modulation signal, respectively, which denote the measured information of Eve under “Attack SNL”. Based on orange trace, the noise power of ${\hat{X}}_{{\hat{a}}_{3}}-{\hat{X}}_{{\hat{b}}_{3}}$ (${\hat{P}}_{{\hat{a}}_{4}}+{\hat{P}}_{{\hat{b}}_{4}}$) for Eve without modulated signal is about 0.089 dB (0.119 dB) above the corresponding SNL at 0 dB. Using conventional entanglement witness, i.e., $\left\langle {\rm{EW}}\right\rangle =\left\langle {\Delta }^{2}({\hat{X}}_{{\hat{a}}_{3}}-{\hat{X}}_{{\hat{b}}_{3}})\right\rangle +\left\langle {\Delta }^{2}({\hat{P}}_{{\hat{a}}_{4}}+{\hat{P}}_{{\hat{b}}_{4}})\right\rangle =\frac{1{0}^{\frac{0.089}{10}}+1{0}^{\frac{0.119}{10}}}{1{0}^{\frac{0}{10}}}\times 0.500\approx 1.024 > 1.000$, indicating the absence of entanglement between ${\hat{a}}_{1}$ and ${\hat{b}}_{1}$^32,33^. If Eve deliberately increases the LO intensity when calibrating SNL, the noise power of the SNL changes from the black traces to red traces. As shown in Fig. 4c and Fig. 4d, according to the conventional entanglement detection scheme, the measured quadrature variances (orange traces) of the to-be-verified modes are below the measured SNL (red traces). Based on red trace, the noise power of “Attack SNL” amplitude (phase) quadrature is about 1.356 dB (1.178 dB) above the corresponding SNL at 0 dB. The measured noise power for the to-be-verified modes is undervalued, and $\left\langle {{\rm{EW}}}_{{\rm{A}}}\right\rangle =\left\langle {\Delta }^{2}({\hat{X}}_{{\hat{a}}_{3}}-{\hat{X}}_{{\hat{b}}_{3}})\right\rangle +\left\langle {\Delta }^{2}({\hat{P}}_{{\hat{a}}_{4}}+{\hat{P}}_{{\hat{b}}_{4}})\right\rangle =(\frac{1{0}^{\frac{0.089}{10}}}{1{0}^{\frac{1.356}{10}}}+\frac{1{0}^{\frac{0.119}{10}}}{1{0}^{\frac{1.178}{10}}})\times 0.500\approx 0.765 < 1.000$, which incorrectly witnesses that ${\hat{a}}_{1}$ and ${\hat{b}}_{1}$ are entangled. In other words, the conventional EW method incorrectly identifies a separable state as an entangled state.

However, in the case of MDIEW, if Eve changed the intensity of LO, the previously calibrated noise power will change. As shown in Fig. 4c (Fig. 4d), based on pink trace, the noise power of ${\hat{X}}_{{\hat{a}}_{3}}-{\hat{X}}_{{\hat{b}}_{3}}$ (${\hat{P}}_{{\hat{a}}_{4}}+{\hat{P}}_{{\hat{b}}_{4}}$) for Eve with modulated signal is about 11.723 dB (6.374 dB) above the corresponding SNL at 0 dB. Therefore, the value of $\left\langle {\hat{X}}_{{\hat{a}}_{3}}-{\hat{X}}_{{\hat{b}}_{3}}\right\rangle$ $\langle{\hat{P}}_{{\hat{a}}_{4}}+{\hat{P}}_{{\hat{b}}_{4}}\rangle$ can be calculated as $\frac{1{0}^{\frac{11.723}{10}}-1{0}^{\frac{0.089}{10}}}{1{0}^{\frac{1.356}{10}}}\times 0.500\approx 5.067$ ($\frac{1{0}^{\frac{6.374}{10}}-1{0}^{\frac{0.119}{10}}}{1{0}^{\frac{1.178}{10}}}\times 0.500\approx 1.262$), which results in Eve’s measurement result $\left\langle {\hat{X}}_{{\hat{a}}_{3}}-{\hat{X}}_{{\hat{b}}_{3}}\right\rangle$ ($\left\langle {\hat{P}}_{{\hat{a}}_{4}}+{\hat{P}}_{{\hat{b}}_{4}}\right\rangle$) being inconsistent with the users’ (Alice and Cindy) calibrated coherent state value $\frac{\left\langle {\alpha }_{{1}_{X}}-{\beta }_{{1}_{X}}\right\rangle }{\sqrt{2}}$ $\left(\frac{\left\langle {\alpha }_{{1}_{P}}+{\beta }_{{1}_{P}}\right\rangle }{\sqrt{2}}\right)$. In this sense, based on the measurement and Gaussian fit results, $\left\langle {\rm{MDIEW}}\right\rangle =\left\langle {U}^{2}\right\rangle +\left\langle {V}^{2}\right\rangle =\left\langle {({\hat{X}}_{{\hat{a}}_{3}}-{\hat{X}}_{{\hat{b}}_{3}}-\frac{{\alpha }_{{1}_{X}}-{\beta }_{{1}_{X}}}{\sqrt{2}})}^{2}\right\rangle +\left\langle {({\hat{P}}_{{\hat{a}}_{4}}+{\hat{P}}_{{\hat{b}}_{4}}-\frac{{\alpha }_{{1}_{P}}+{\beta }_{{1}_{P}}}{\sqrt{2}})}^{2}\right\rangle =4.773\pm 0.045 > 0.990$, which makes the left-hand value of the Eq. (3) too large to violate Eq. (3). This indicates that there is no entanglement between ${\hat{a}}_{1}$ and ${\hat{b}}_{1}$, which is consistent with the fact. The MDIEW method can reliably verify entanglement even when the intensity of the LO beam is changed. The primary objective of the MDIEW protocol is to ensure that separable states cannot be falsely identified as entangled states even if the measurement device is untrustworthy^31^.

**Fig. 5:**
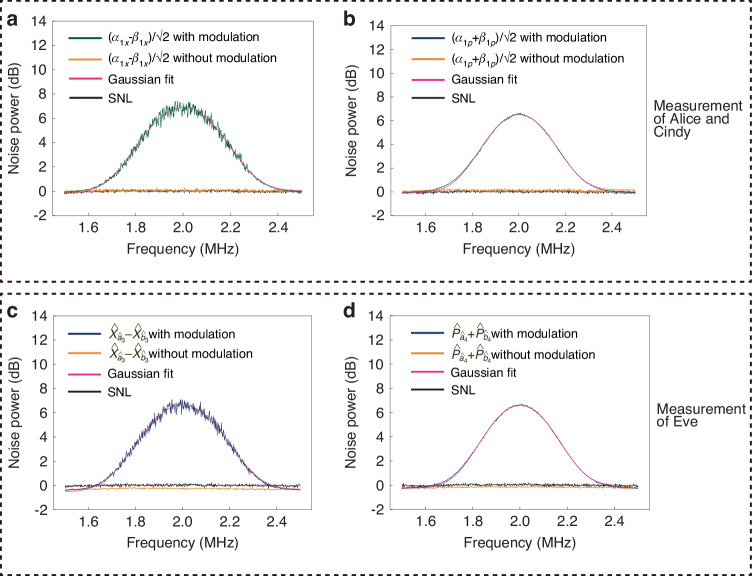
MDIEW in quantum network. **a** and **b** are typical noise power results measured by Alice and Cindy. **c** and **d** are typical noise power results measured by Eve

The MDIEW is used to detect entanglement in our quantum network. The typical measurement results of detecting entanglement between ${\hat{a}}_{1}$ and ${\hat{b}}_{1}$ are shown in Fig. 5. All black traces at 0 dB represent the normalized SNLs. The peak of green trace and orange trace in Fig. 5a (Fig. 5b) at about 2 MHz represent the measurement results of $\frac{{\alpha }_{{1}_{X}}-{\beta }_{{1}_{X}}}{\sqrt{2}}$ ($\frac{{\alpha }_{{1}_{P}}+{\beta }_{{1}_{P}}}{\sqrt{2}}$) for Alice and Cindy with and without modulation signal, respectively. As shown in Fig. 5a (Fig. 5b), based on pink trace, the noise power of $\frac{{\alpha }_{{1}_{X}}-{\beta }_{{1}_{X}}}{\sqrt{2}}$ ($\frac{{\alpha }_{{1}_{P}}+{\beta }_{{1}_{P}}}{\sqrt{2}}$) for Alice and Cindy with modulated signal is about 6.870 dB (6.478 dB) above the corresponding SNL at 0 dB. Based on orange trace, the noise power of $\frac{{\alpha }_{{1}_{X}}-{\beta }_{{1}_{X}}}{\sqrt{2}}$ ($\frac{{\alpha }_{{1}_{P}}+{\beta }_{{1}_{P}}}{\sqrt{2}}$) for Alice and Cindy without modulated signal is about 0.068 dB (0.097 dB) above the corresponding SNL at 0 dB. Therefore, the value of $\frac{\left\langle {\alpha }_{{1}_{X}}-{\beta }_{{1}_{X}}\right\rangle }{\sqrt{2}}$ $\left(\frac{\left\langle {\alpha }_{{1}_{P}}+{\beta }_{{1}_{P}}\right\rangle }{\sqrt{2}}\right)$ can be calculated as $\frac{1{0}^{\frac{6.870}{10}}-1{0}^{\frac{0.068}{10}}}{1{0}^{\frac{0}{10}}}\times 0.500\approx 1.924$ ($\frac{1{0}^{\frac{6.478}{10}}-1{0}^{\frac{0.097}{10}}}{1{0}^{\frac{0}{10}}}\times 0.500\approx 1.711$). The peak of blue trace and orange trace in Fig. 5c (Fig. 5d) at about 2 MHz represent the measurement results of ${\hat{X}}_{{\hat{a}}_{3}}-{\hat{X}}_{{\hat{b}}_{3}}$ (${\hat{P}}_{{\hat{a}}_{4}}+{\hat{P}}_{{\hat{b}}_{4}}$) for Eve with and without modulation signal, respectively. As shown in Fig. 5c (Fig. 5d), based on pink trace, the noise power of ${\hat{X}}_{{\hat{a}}_{3}}-{\hat{X}}_{{\hat{b}}_{3}}$ (${\hat{P}}_{{\hat{a}}_{4}}+{\hat{P}}_{{\hat{b}}_{4}}$) for Eve with modulated signal is about 6.602 dB (6.548 dB) above the corresponding SNL at 0 dB. Based on orange trace, the noise power of ${\hat{X}}_{{\hat{a}}_{3}}-{\hat{X}}_{{\hat{b}}_{3}}$ (${\hat{P}}_{{\hat{a}}_{4}}+{\hat{P}}_{{\hat{b}}_{4}}$) for Eve without modulated signal is about 0.293 dB (0.189 dB) below the corresponding SNL at 0 dB. Therefore, the value of $\left\langle {\hat{X}}_{{\hat{a}}_{3}}-{\hat{X}}_{{\hat{b}}_{3}}\right\rangle$ $\left(\left\langle {\hat{P}}_{{\hat{a}}_{4}}+{\hat{P}}_{{\hat{b}}_{4}}\right\rangle\right)$ can be calculated as $\frac{1{0}^{\frac{6.602}{10}}-1{0}^{\frac{-0.293}{10}}}{1{0}^{\frac{0}{10}}}\times 0.500\approx 1.819$ ($\frac{1{0}^{\frac{6.548}{10}}-1{0}^{\frac{-0.189}{10}}}{1{0}^{\frac{0}{10}}}\times 0.500\approx 1.780$), the value of $Var({\hat{X}}_{{\hat{a}}_{3}}-{\hat{X}}_{{\hat{b}}_{3}})$ [$Var({\hat{P}}_{{\hat{a}}_{4}}+{\hat{P}}_{{\hat{b}}_{4}})$] can be calculated as $\frac{1{0}^{\frac{-0.293}{10}}}{1{0}^{\frac{0}{10}}}\times 0.500\approx 0.467$ ($\frac{1{0}^{\frac{-0.189}{10}}}{1{0}^{\frac{0}{10}}}\times 0.500\approx 0.479$). By substituting the above calculation results into Eqs. (3) and (4), $\langle {\rm{MDIEW}}\rangle =\langle {U}^{2}\rangle +\langle {V}^{2}\rangle =\langle {({\hat{X}}_{{\hat{a}}_{3}}-{\hat{X}}_{{\hat{b}}_{3}}-\frac{{\alpha }_{{1}_{X}}-{\beta }_{{1}_{X}}}{\sqrt{2}})}^{2}\rangle +\langle {({\hat{P}}_{{\hat{a}}_{4}}+{\hat{P}}_{{\hat{b}}_{4}}-\frac{{\alpha }_{{1}_{P}}+{\beta }_{{1}_{P}}}{\sqrt{2}})}^{2}\rangle =0.962\pm 0.016 < 0.990$, which violates Eq. (3). This verifies that there is entanglement between ${\hat{a}}_{1}$ and ${\hat{b}}_{1}$. The experimental results of entanglement between ${\hat{a}}_{1}$ and ${\hat{b}}_{2},{\hat{a}}_{2}$ and ${\hat{b}}_{1},{\hat{a}}_{2}$ and ${\hat{b}}_{2}$ are similar to the results above (see “2. The experimental entanglement detection results with different users” and Figs. S2, S3 and S4 in Supplementary Information for details). These results demonstrate that we have verified the entanglements in a four-user quantum network.

## Discussion

We have experimentally demonstrated the MDIEW in a multi-user CV quantum network. When the measured noise power during calibrating SNL of the measurement device is changed, conventional EW method may give unreliable results. In our quantum network, four pairs of EPR entangled modes between four optical modes ({${\hat{a}}_{1},{\hat{b}}_{1}$}, {${\hat{a}}_{1},{\hat{b}}_{2}$}, {${\hat{a}}_{2},{\hat{b}}_{1}$}, and {${\hat{a}}_{2},{\hat{b}}_{2}$}) are reliably verified based on the MDIEW method. Our results provide a trustworthy method to detect CV entanglement, which can potentially add further security to the quantum secret sharing network^19^.

## Materials and methods

### Theoretical squeezing between entangled modes

In the experiment, a pair of EPR entangled modes $\hat{a}$ and $\hat{b}$ are generated based on the FWM process in hot ^85^Rb atomic vapor cell. Mode $\hat{a}$ ($\hat{b}$) is divided into modes ${\hat{a}}_{1}$ (${\hat{b}}_{1}$) and ${\hat{a}}_{2}$ (${\hat{b}}_{2}$) by the 50:50 BS, thereby generating entangled resources for a four-user quantum network. In the MDIEW protocol, the to-be-verified mode (${\hat{a}}_{1},{\hat{b}}_{1},{\hat{a}}_{2},{\hat{b}}_{2}$) and the corresponding coherent state ($\left\vert {\alpha }_{1}\right\rangle ,\left\vert {\beta }_{1}\right\rangle ,\left\vert {\alpha }_{2}\right\rangle ,\left\vert {\beta }_{2}\right\rangle$) prepared by the user are also combined by the 50:50 BSs. Therefore, for measuring the to-be-verified mode (${\hat{a}}_{1},{\hat{b}}_{1},{\hat{a}}_{2},{\hat{b}}_{2}$) in MDIEW, the above BSs introduce a loss of *η* ≈ 0.75 to the initial modes $\hat{a}$ and $\hat{b}$, introducing vacuum states (${\hat{v}}_{1},{\hat{v}}_{2},{\hat{v}}_{3},{\hat{v}}_{4}$). Besides, we also model the absorption *η*_*a*_ (*η*_*b*_) of mode $\hat{a}$ ($\hat{b}$) from ^85^Rb atomic vapor cell and other optical devices through another BS which introduces a vacuum state ${\hat{v}}_{5}$ (${\hat{v}}_{6}$). Considering all these losses, based on Eq. (1), the input-output relationship for the to-be-verified mode can be expressed as

5 $$\begin{array}{ll}{\hat{a}}_{1}=\sqrt{(1-\eta )(1-{\eta}_{a})G}{\hat{a}}_{0}+\sqrt{(1-\eta )(1-{\eta}_{a})(G-1)}{\hat{b}}_{0}^{\dagger }\\\quad +\sqrt{(1-\eta ){\eta}_{a}}{\hat{v}}_{5}+\sqrt{\eta }{\hat{v}}_{1}\\ {\hat{b}}_{1}=\sqrt{(1-\eta )(1-{\eta }_{b})G}{\hat{b}}_{0}+\sqrt{(1-\eta )(1-{\eta}_{b})(G-1)}{\hat{a}}_{0}^{\dagger }\\\quad +\sqrt{(1-\eta ){\eta}_{b}}{\hat{v}}_{6}+\sqrt{\eta}{\hat{v}}_{2}\\ {\hat{a}}_{2}=\sqrt{(1-\eta )(1-{\eta }_{a})G}{\hat{a}}_{0}+\sqrt{(1-\eta )(1-{\eta}_{a})(G-1)}{\hat{b}}_{0}^{\dagger }\\\quad +\sqrt{(1-\eta ){\eta}_{a}}{\hat{v}}_{5}+\sqrt{\eta }{\hat{v}}_{3}\\ {\hat{b}}_{2}=\sqrt{(1-\eta )(1-{\eta }_{b})G}{\hat{b}}_{0}+\sqrt{(1-\eta )(1-{\eta}_{b})(G-1)}{\hat{a}}_{0}^{\dagger }\\\quad +\sqrt{(1-\eta ){\eta}_{b}}{\hat{v}}_{6}+\sqrt{\eta }{\hat{v}}_{4}\end{array}$$

The theoretical squeezing between mode ${\hat{a}}_{1}$ and ${\hat{b}}_{1}$ (${\hat{a}}_{1}$ and ${\hat{b}}_{2},{\hat{a}}_{2}$ and ${\hat{b}}_{1},{\hat{a}}_{2}$ and ${\hat{b}}_{2}$) can be expressed as

6 $$10\,{\log}_{10}\{1+(1-\eta)\left[(G-1)(2-{\eta }_{a}-{\eta}_{b})-2\sqrt{(1-{\eta}_{a})(1-{\eta }_{b})G(G-1)}\right]\}$$

In the experiment, *G* ≈ 1.23, *η*_*a*_ ≈ 0.20, *η*_*b*_ ≈ 0.15, *η* ≈ 0.75. This results in only 0.58 dB squeezing remaining between entangled modes ${\hat{a}}_{1}$ and ${\hat{b}}_{1}$ (${\hat{a}}_{1}$ and ${\hat{b}}_{2},{\hat{a}}_{2}$ and ${\hat{b}}_{1},{\hat{a}}_{2}$ and ${\hat{b}}_{2}$) theoretically. In addition, the imperfect phase locking also introduces some extra noise into the system, which further attenuates the entanglement strength^34^. As a result, the entanglement observed in our setup is relatively weak.

## Supplementary information

Supplementary file for Measurement-Device-Independent Continuous Variable Entanglement Witness in a Quantum Network

## Data Availability

The data that support the findings of this study are available from the corresponding author upon reasonable request.
