# Supplementary material for: Measurement-device-independent continuous variable entanglement witness in a quantum network
Source: Light Sci Appl. 2025 Nov 3;14:376. doi: 10.1038/s41377-025-02039-x (PMC12580416; doi:10.1038/s41377-025-02039-x)
Supplement: Supplementary file 1 — Supplementary file for Measurement-Device-Independent Continuous Variable Entanglement Witness in a Quantum Network [file 41377_2025_2039_MOESM1_ESM.pdf]

Supplementary Information for

Measurement-Device-Independent Continuous Variable Entanglement Witness in a Quantum Network

Jing Fu[1†], Xutong Wang[1†], Shengshuai Liu[1*], and Jietai Jing[1,2,3*]
[1]State Key Laboratory of Precision Spectroscopy, Joint Institute of Advanced Science and Technology, School of Physics and Electronic Science, East China Normal University, Shanghai 200062, China
[2]Collaborative Innovation Center of Extreme Optics, Shanxi University, Taiyuan, Shanxi 030006, China
[3]CAS Center for Excellence in Ultra-intense Laser Science, Shanghai, 201800, China
[†]These authors contributed equally to this work
*Corresponding author: *ssliu@lps.ecnu.edu.cn* (Shengshuai Liu), *jtjing@phy.ecnu.edu.cn* (Jietai Jing)

1. Selection of parameter σ value

The parameter σ determines the width of the Gaussian distribution used to randomly choose the coherent states, and it directly affects the bound of the witness inequality and thus the success of entanglement witness. As mentioned in Ref. [17], measurement device independent entanglement witness (MDIEW) can be expressed as

$$\langle \mathrm{MDIEW} \rangle = \frac{1}{2}(1 + e^{-2r}) \geq \frac{\sigma^2}{1+\sigma^2}, \tag{S1}$$

where r is squeezing parameter. If Eq. (S1) is violated, it means that there is entanglement between the witnessed modes. As shown in Fig. S1, the black trace represents the corresponding relationship between the $\langle \mathrm{MDIEW} \rangle$ value and r. The orange, green, blue, purple, and pink traces in Fig. S1 represent the $\frac{\sigma^2}{1+\sigma^2}$ values of $\sigma = 2, 4, 6, 8$, and 10, respectively. As mentioned in Ref. [17], for a specific squeezing parameter r, the parameter σ should be large enough to violate Eq. (S1). In our scheme, eight sets of experimentally measured squeezing r (0.040, 0.045, 0.030, 0.029, 0.059, 0.031, 0.066, 0.046) are marked with red pentagrams in Fig. S1. In this case, parameter σ should be larger than 6 to ensure the violation of MDIEW inequality. Therefore, it is reasonable to select $\sigma = 10$ in our scheme.

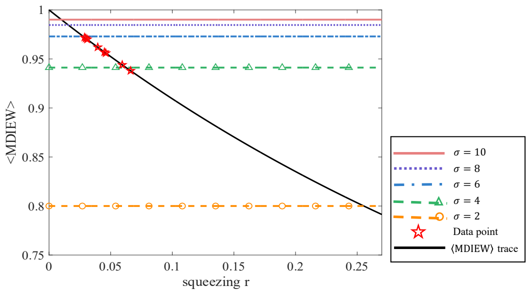

Fig. S1 The relationship between the ⟨MDIEW⟩ value and r. The black trace represents relationship between the ⟨MDIEW⟩ value and r. The orange, green, blue, purple, and pink traces represent the $\frac{\sigma^2}{1+\sigma^2}$ values of $\sigma = 2, 4, 6, 8$, and 10, respectively, where σ is the width of the Gaussian distribution used to randomly choose the coherent states. The red pentagrams represent our experimental measured squeezing.

2. The experimental entanglement detection results with different users

Alice and Cindy prepare another different set of trusted and well-calibrated coherent states to detect the entanglement between $\hat{a}_1$ and $\hat{b}_1$, respectively. Based on the measurement and Gaussian fit results, $Var(\hat{X}_{\hat{a}_3} - \hat{X}_{\hat{b}_3}) = 0.465$, $Var(\hat{P}_{\hat{a}_4} + \hat{P}_{\hat{b}_4}) = 0.484$, $\langle MDIEW \rangle = \langle \left(\hat{X}_{\hat{a}_3} - \hat{X}_{\hat{b}_3} - \frac{\alpha_{1X} - \beta_{1X}}{\sqrt{2}}\right)^2 \rangle + \langle \left(\hat{P}_{\hat{a}_4} + \hat{P}_{\hat{b}_4} - \frac{\alpha_{1P} + \beta_{1P}}{\sqrt{2}}\right)^2 \rangle = 0.957 \pm 0.016 < 0.990$, which violates Eq. (3) in main manuscript.

Alice and David prepare two different sets of trusted and well-calibrated coherent states to detect the entanglement between $\hat{a}_1$ and $\hat{b}_2$, respectively. Using one set, the typical results of the entanglement detection between $\hat{a}_1$ and $\hat{b}_2$ by the MDIEW are shown in Fig. S2. Based on the measurement and Gaussian fit results, $Var(\hat{X}_{\hat{a}_3} - \hat{X}_{\hat{b}_5}) = 0.475$, $Var(\hat{P}_{\hat{a}_4} + \hat{P}_{\hat{b}_6}) = 0.478$, $\langle MDIEW \rangle = \langle \left(\hat{X}_{\hat{a}_3} - \hat{X}_{\hat{b}_5} - \frac{\alpha_{1X} - \beta_{2X}}{\sqrt{2}}\right)^2 \rangle + \langle \left(\hat{P}_{\hat{a}_4} + \hat{P}_{\hat{b}_6} - \frac{\alpha_{1P} + \beta_{2P}}{\sqrt{2}}\right)^2 \rangle = 0.971 \pm 0.016 < 0.990$, which violates Eq. (3) in main manuscript. Using the other set, based on the measurement and Gaussian fit results, $Var(\hat{X}_{\hat{a}_3} - \hat{X}_{\hat{b}_5}) = 0.479$, $Var(\hat{P}_{\hat{a}_4} + \hat{P}_{\hat{b}_6}) = 0.481$, $\langle MDIEW \rangle = \langle \left(\hat{X}_{\hat{a}_3} - \hat{X}_{\hat{b}_5} - \frac{\alpha_{1X} - \beta_{2X}}{\sqrt{2}}\right)^2 \rangle + \langle \left(\hat{P}_{\hat{a}_4} + \hat{P}_{\hat{b}_6} - \frac{\alpha_{1P} + \beta_{2P}}{\sqrt{2}}\right)^2 \rangle = 0.972 \pm 0.016 < 0.990$, which violates Eq. (3) in main manuscript. These results show that there is entanglement between $\hat{a}_1$ and $\hat{b}_2$.

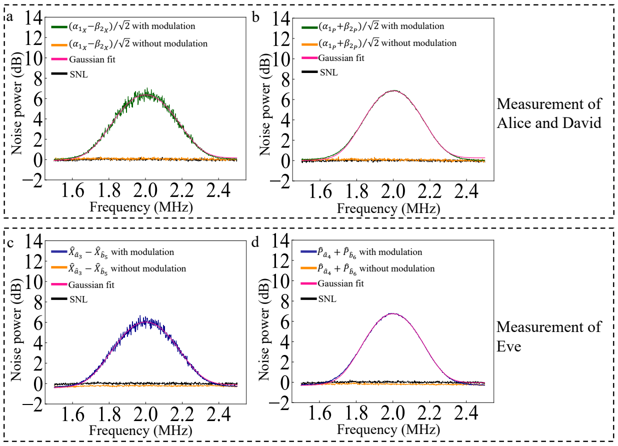

Fig. S2. (a) and (b) are typical noise power results measured by Alice and David. (c) and (d) are typical noise power results measured by Eve.

Bob and Cindy prepare two different sets of trusted and well-calibrated coherent states to detect the entanglement between $\hat{a}_2$ and $\hat{b}_1$, respectively. Using one set, the typical results of the entanglement detection between $\hat{a}_2$ and $\hat{b}_1$ by the MDIEW are shown in Fig. S3. Based on the measurement and Gaussian fit results, $Var(\hat{X}_{\hat{a}_5} - \hat{X}_{\hat{b}_3}) = 0.469$, $Var(\hat{P}_{\hat{a}_6} + \hat{P}_{\hat{b}_4}) = 0.468$, $\langle MDIEW \rangle = \langle \left(\hat{X}_{\hat{a}_5} - \hat{X}_{\hat{b}_3} - \frac{\alpha_{2X} - \beta_{1X}}{\sqrt{2}}\right)^2 \rangle + \langle \left(\hat{P}_{\hat{a}_6} + \hat{P}_{\hat{b}_4} - \frac{\alpha_{2P} + \beta_{1P}}{\sqrt{2}}\right)^2 \rangle = 0.944 \pm 0.017 < 0.990$, which violates Eq. (3) in main manuscript. Using the other set, based on the measurement and Gaussian fit results, $Var(\hat{X}_{\hat{a}_5} - \hat{X}_{\hat{b}_3}) = 0.477$, $Var(\hat{P}_{\hat{a}_6} + \hat{P}_{\hat{b}_4}) = 0.479$, $\langle MDIEW \rangle = \langle \left(\hat{X}_{\hat{a}_5} - \hat{X}_{\hat{b}_3} - \frac{\alpha_{2X} - \beta_{1X}}{\sqrt{2}}\right)^2 \rangle + \langle \left(\hat{P}_{\hat{a}_6} + \hat{P}_{\hat{b}_4} - \frac{\alpha_{2P} + \beta_{1P}}{\sqrt{2}}\right)^2 \rangle = 0.970 \pm 0.016 < 0.990$, which violates Eq. (3) in main manuscript. These results show that there is entanglement between $\hat{a}_2$ and $\hat{b}_1$.

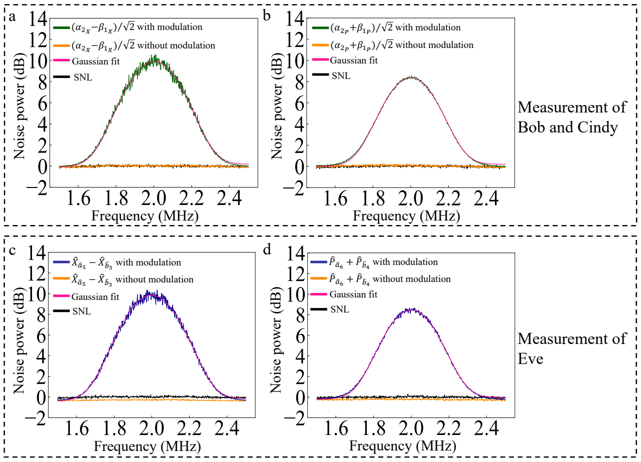

Fig. S3. (a) and (b) are typical noise power results measured by Bob and Cindy. (c) and (d) are typical noise power results measured by Eve.

Bob and David prepare two different sets of trusted and well-calibrated coherent states to detect the entanglement between $\hat{a}_2$ and $\hat{b}_2$, respectively. Using one set, the typical results of the entanglement detection between $\hat{a}_2$ and $\hat{b}_2$ by the MDIEW are shown in Fig. S4. Based on the measurement and Gaussian fit results, $Var(\hat{X}_{\hat{a}_5} - \hat{X}_{\hat{b}_5}) = 0.464$, $Var(\hat{P}_{\hat{a}_6} + \hat{P}_{\hat{b}_6}) = 0.472$, $\langle MDIEW \rangle = \langle \left(\hat{X}_{\hat{a}_5} - \hat{X}_{\hat{b}_5} - \frac{\alpha_{2X} - \beta_{2X}}{\sqrt{2}}\right)^2 \rangle + \langle \left(\hat{P}_{\hat{a}_6} + \hat{P}_{\hat{b}_6} - \frac{\alpha_{2P} + \beta_{2P}}{\sqrt{2}}\right)^2 \rangle = 0.938 \pm 0.017 < 0.990$, which violates Eq. (3) in main manuscript. Using the other set, based on the measurement and Gaussian fit results, $Var(\hat{X}_{\hat{a}_5} - \hat{X}_{\hat{b}_5}) = 0.478$, $Var(\hat{P}_{\hat{a}_6} + \hat{P}_{\hat{b}_6}) = 0.475$, $\langle MDIEW \rangle = \langle \left(\hat{X}_{\hat{a}_5} - \hat{X}_{\hat{b}_5} - \frac{\alpha_{2X} - \beta_{2X}}{\sqrt{2}}\right)^2 \rangle + \langle \left(\hat{P}_{\hat{a}_6} + \hat{P}_{\hat{b}_6} - \frac{\alpha_{2P} + \beta_{2P}}{\sqrt{2}}\right)^2 \rangle = 0.956 \pm 0.017 < 0.990$, which violates Eq. (3) in main manuscript. These results show that there is entanglement between $\hat{a}_2$ and $\hat{b}_2$. In our scheme, with different modulation signals, the values of ⟨MDIEW⟩ for witnessing the same modes are slightly different due to the imperfection of electro-optic modulator.

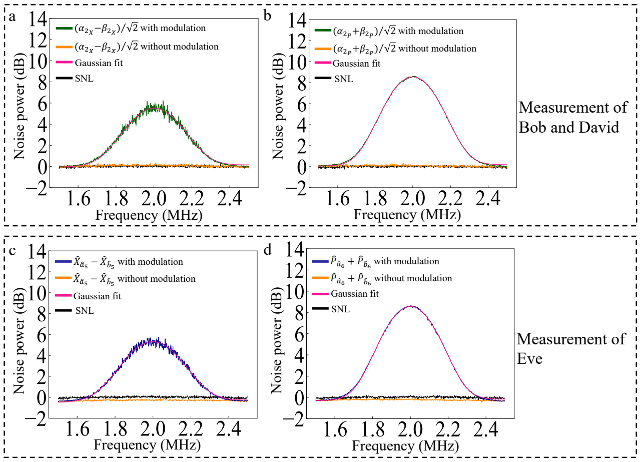

Fig. S4. (a) and (b) are typical noise power results measured by Bob and David. (c) and (d) are typical noise power results measured by Eve.
